# Behavior Management Training for Parents of Children with Preschool ADHD Based on Parent-Child Interactions: A Multicenter Randomized Controlled, Follow-Up Study

**DOI:** 10.1155/2023/3735634

**Published:** 2023-09-11

**Authors:** Min Feng, Juncai Xu, Mengyao Zhai, Qiaorong Wu, Kangkang Chu, Liping Xie, Rong Luo, Huiping Li, Qiong Xu, Xiu Xu, Xiaoyan Ke

**Affiliations:** ^1^Nanjing Rehabilitation Medical Center, The Affiliated Brain Hospital of Nanjing Medical University, Nanjing 210029, China; ^2^School of Engineering, Case Western Reserve University, Cleveland, OH 44106, USA; ^3^West China Second University Hospital, Sichuan University, Chengdu 610041, China; ^4^Children's Hospital of Fudan University, Shanghai 20110, China

## Abstract

**Objective:**

There is a need to develop optimized, evidence-based parent training programs tailored for preschoolers with attention deficit hyperactivity disorder (ADHD). The objective of this study was to explore a behavioral management training program aimed at the parents of preschool children with ADHD, which directly analyzes parent-child interaction from the perspective of system theory, and the intervention effect on ADHD in preschool children.

**Methods:**

A multicenter randomized controlled study was conducted using system-based group therapy with 62 parents of preschool children with ADHD aged four to six years. ADHD symptoms, behavioral and emotional problems, and social functioning were compared with 61 control children whose parents did not receive training by applying the ADHD Rating Scale (ADHD-RS), Strengths and Difficulties Questionnaire (SDQ), and Questionnaire-Children with Difficulties (QCD) at the time of subject entry and at two and six months of entry, respectively.

**Results:**

The results of the ADHD-RS assessment showed that children in the intervention group had significantly lower factor scores for attention deficit, hyperactivity, and impulsivity than the children in the control group after parental training and at follow-up (*P* < 0.05). Total scores on the SDQ scale, as well as character problems, hyperactivity, and peer interaction scores, significantly decreased with statistically significant differences (all *P* < 0.05), and emotional symptoms and prosocial behavior did not notable decline (*P* > 0.05). Compared with the control group, the total scores of the QCD scale and the scores of each factor in the intervention group remained significantly higher at the follow-up (*P* < 0.05).

**Conclusion:**

After continuous intervention for eight weeks, parents were able to help the children with preschool ADHD to improve their ADHD symptoms and emotional behavioral and social functioning significantly, and the efficacy was maintained at the four-month follow-up; the systemic-based parent training in behavior management (PTBM) is applicable to the treatment of preschool ADHD and is worth promoting.

## 1. Introduction

Attention deficit hyperactivity disorder (ADHD), the most common psychiatric disorder in children, is characterized by inattention, hyperactivity, and impulsivity [1]. ADHD at preschool age is as common as at school age, with a prevalence of 2%–5% [2, 3]. In addition, 65% to 89% of preschoolers with ADHD-like symptoms continue to have ADHD symptoms during school age and still meet the diagnostic criteria [4, 5]. Studies of ADHD symptom trajectories have shown that ADHD symptoms develop gradually. ADHD-like symptoms in preschoolers predict an elevated risk of distant academic and social difficulties and cooccurring emotional and behavioral problems [5–7]. ADHD, as a neurodevelopmental disorder distinguished by impulsivity or hyperactivity, severely interferes with the social, emotional, and cognitive levels of children in their natural developmental environment [8], significantly impacting the life, academic, social, and family functioning of affected children. Evidence has shown that children with preschool ADHD are at higher risk for cooccurring oppositional defiant disorder (ODD), conduct disorder (CD), and anxiety and depression problems [9, 10] and are more likely to develop disruptive behaviors and substance abuse with increasing age, leading to severe consequences [11]. The earlier the onset of ADHD, the greater the risk of other associated occurrences [12]. Given the severe consequences of ADHD, it is clear that early and effective interventions are essential for its treatment.

The neuroplasticity in young children is notably higher compared to that of older children [13]. This enhanced plasticity signifies an amplified capacity to form novel neural connections and to remodel themselves in response to various experiences and interventions [14]. Importantly, plasticity reaches its zenith during early childhood, a pivotal period marked by swift brain development and pronounced adaptability [15]. This decisive phase presents a distinctive opportunity for interventions, such as those targeting conditions like ADHD, to instigate profound and enduring effects on disease progression, given the concurrent evolution of the brain's functional networks [16]. Studies suggest that initiating behavioral interventions at this stage, such as structured parental training, can strengthen neural circuits involved in self-regulation [17]. Beyond merely rectifying ADHD-associated neural dysfunctions, applying interventions during this period of heightened plasticity can forestall potential detrimental alterations in brain function that may manifest progressively over time [18]. Therefore, early intervention and treatment may yield lasting benefits and improve unfavorable disease trajectories [19]. In addition to reducing symptom severity, it may also prevent problems associated with ADHD, such as peer rejection and low self-esteem, and reduce the risk of cooccurring ODD/CD [13, 20, 21]. ADHD can be treated with medication, but its efficacy is less consistent [22]. It is also associated with more adverse effects in preschool children [23]. Parent training in behavior management (PTBM) for parents (or primary caregivers) is the first line of treatment for preschool ADHD [24–26]. Existing research and clinical practice have demonstrated that PTBM for preschool children with ADHD can alleviate core ADHD symptoms; improve parent-child relationships, peer relationships, and the quality of life; and reduce the incidence of comorbidities [27, 28].

Despite the effectiveness of preschool PTBM, continued attention to behavioral programs is essential because the diversity of interventions and outcome measures makes the effects of behavioral treatment variable whether alone or with medication [25]. Developing evidence-based intervention programs for preschool PTBM has received increasing attention in recent years. The main intervention programs available for preschool PTBM include the Positive Parenting Program (Triple P) [29], New Forest Parenting Programme (NFPP) [27], Incredible Years [28], and parent-child interaction therapy (PCIT) [30]. The content of these programs varies considerably, for example, the NFPP combines traditional parent training with some parent-child interaction elements designed to specifically address the underlying processes associated with ADHD [30]; PCIT proposes an intervention approach for targeting the parent-child interaction model [31]; Triple P is a parent training approach designed to prevent serious behavioral, emotional, and developmental problems in children by improving parents' illness awareness, skills, and self-confidence [32]; and Incredible Years focuses on positive reinforcement [33]. However, most behavioral parent training programs are rooted in social learning theory [34]. Moreover, they are mainly based on attachment theory in changing parent-child relationships, with less direct analysis of parent-child interactions at the behavioral level from the perspective of systems theory. The systems theory perspective emphasizes analyzing the interactions and bidirectional influences between various elements within a system, rather than viewing behavior as the product of isolated causal factors. In the context of parent-child interactions, the systems theory perspective highlights that both parent and child behaviors mutually influence each other in an ongoing, reciprocal process. In contrast, attachment theory focuses on how the parent-child bond impacts child development and behavior. While important, it does not directly analyze the real-time, behavioral dynamics between parent and child. Social learning theory examines how behaviors are learned through observation, reinforcement, etc. but does not focus on the interactive system of parent-child dyads.

This study, integrating the effective elements of existing training programs, proposes a preschool PTBM intervention program that considers the occurrence, development, and influencing factors of ADHD symptoms from a systems theory perspective. This program directly analyzes the bidirectional influences between parents and children during the implementation of interventions at the behavioral level. By instructing parents on how to effectively dissect and understand the reciprocal dynamics within parent-child interactions, we can cultivate their emotional awareness, acceptance, and emotional regulation capabilities. This empowerment allows parents to adopt smarter, more thoughtful intervention strategies when managing their children's behavioral challenges.

Therefore, this study conducted a multicenter study of parent group efficacy. The study combined five units across different sites. Offline and online training was provided to the therapists and physicians implementing the intervention at each center to ensure that the interventionists had an accurate grasp of parent training strategies. Regular supervision was conducted to ensure the accuracy and consistency of implementation. In addition, a detailed operation manual was developed and distributed to the intervention staff carrying out the parent training to ensure consistency. Each center conducted a randomized control, and to ensure data safety and objectivity, an observer-blind strategy was adopted, with those implementing the intervention separated from those distributing the collected data. The main contributions of this paper are as follows.

- We propose a behavioral analysis based on parent-child interactions emphasizing parental emotional awareness and management to ensure that parents are empowered to implement child-specific behavior management
- It represents China's first multicenter study to conduct behavior management training for parents of children with preschool ADHD
- It is a large-sample, randomized controlled, and follow-up study

This study proposes a training intervention program involving parent-child interaction for preschool ADHD, which provides a practical pathway for intervention treatment of preschool ADHD. First, this paper briefly describes the efficacy of parent group therapy in improving ADHD core symptoms and emotional behavioral and social functioning in preschool ADHD. Then, in Section 2, we describe this intervention protocol in detail, as well as the study process and statistical analyses. Next, the results are presented in Section 3. Finally, the results of this study are analyzed and discussed in Section 4, and the conclusions are outlined in Section 5.

## 2. Materials and Methods

### 2.1. Study Design

**Figure 1:**
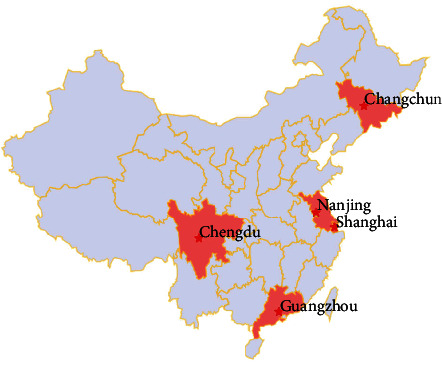
Regional distribution of multicenter randomized controlled studies.

This investigation was a multicenter, randomized controlled study involving five study centers, including the Nanjing Brain Hospital, Children's Hospital of Fudan University, Second Hospital of West China of Sichuan University, Guangzhou Women and Children's Medical Center, and First Hospital of Jilin University (Figure 1). Prior to this study, a pilot study was conducted from January to September 2018, with a total of 36 participants, 15 in the intervention group and 11 in the control group. The pilot study aided us in refining the intervention plan and assessment tools. All participants in the intervention and control groups were enrolled from May 2019 to January 2021. Each center recruited eligible parents of preschool children with ADHD through advertisements, referrals, and screenings. Parents were informed about the study's objectives, procedures, potential benefits, and risks prior to participation and signed an informed consent form. These parents were then randomly assigned to either the intervention or control group by researchers who were not involved in the study and unaware of the intervention status using a computer-generated random number system [35]. The randomization and the intervention were conducted independently at each center.

The intervention group participated in offline parental group sessions weekly for eight weeks, while the control group did not participate in any form of parental training program. The implementation of the parental group intervention sessions was orchestrated in a series of phased cohorts at each designated location. This involved the execution of one to two successive cycles, each consisting of eight consecutive weekly sessions, with an involvement of six to ten parents per cycle. Consequently, the comprehensive duration of the intervention process extended over approximately 4 months a year at each respective center. Symptom and function assessments were conducted at the time of enrollment, two months postenrollment, and six months postenrollment. These assessments were carried out by researchers who were blind to group assignment and used standardized tools such as the ADHD Rating Scale (ADHD-RS), Strengths and Difficulties Questionnaire (SDQ), and Questionnaire-Children with Difficulties (QCD). Data collection for all participants who completed the intervention and follow-up assessments was completed in August 2021. Data compilation was completed from August 15 to September 7, 2021.

### 2.2. Intervention Quality Control

In December 2018, we trained a total of 15 interventionists, including therapists and doctors involved in the intervention, both online and offline, averaging three interventionists per center. The training was conducted under the guidance of a senior child psychiatry specialist and two clinical psychology experts, including online lectures, offline seminars, and supervision meetings. The offline training included 12 hours of face-to-face lectures and role-plays, covering the theoretical background, operation manuals, and case examples of parent behavior management training. Online training included eight hours of video seminars and Q&A sessions. From December 2018 to December 2020, one offline training and three online supervisions were completed. Therapists and doctors were eligible for intervention only after completing the offline training. Each supervision meeting required at least two interventionists from each center to participate. The ratio of online to face-to-face training was similar across the five centers, ranging from 70% to 80% for face-to-face training, and from 50% to 70% for online training. Throughout the intervention period, individual guidance and real-time Q&A were provided via working groups to ensure the accuracy of the intervention, ensuring the accuracy and consistency of the intervention implementation.

### 2.3. Subjects and Recruitment

A total of 235 parents of preschool-aged children with ADHD aged four to six years were enrolled in all the study centers from May 2019 to January 2021, with 140 in the intervention group and 95 in the control group. The specific inclusion criteria are described below.

**Figure 2:**
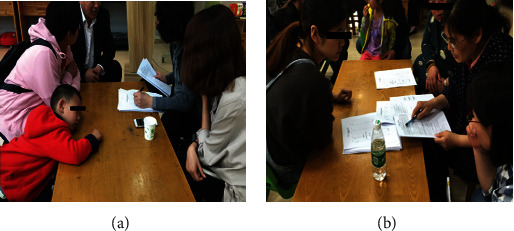
Example of subject recruitment site: (a) subject 1 and (b) subject 2.

The intervention group entry criteria included (i) diagnosis by a child psychiatry clinical specialist according to the *Diagnostic and Statistical Manual of Mental Disorders, Fifth Edition* (DSM⁃5) diagnostic criteria and a brief international neuropsychiatric interview for children and adolescents (MINI Kid) [36]. The children met the DSM-5 diagnostic criteria for ADHD and had no other organic diseases, (ii) were four to six years old, and (iii) had a Wechsler IQ ≥ 80, while the parents (iv) were the primary caregivers living with the child and (v) voluntarily received systematic parent training and completed the follow-up. The control group had the same diagnostic criteria, age, and IQ as the intervention group. They voluntarily participated and completed the full follow-up (Figure 2).

### 2.4. Research Tools

#### 2.4.1. ADHD Rating Scale (ADHD-RS)

The ADHD-RS is the corresponding 18-item ADHD symptom scale in the DSM-IV, of which nine are attention deficit symptoms, and nine are hyperactive-impulsive symptoms. The internal consistency is high, with Cronbach's alpha around 0.92 for the total score. Test-retest reliability over 4 weeks is 0.85. It also shows good convergent and divergent validity when correlated with other ADHD assessments. The frequency of symptoms was evaluated on a 4-point scale from 0 (none) to 3 (always), and the total scale score, attention deficit score, and hyperactivity subscale score were used as indicators of efficacy [37].

#### 2.4.2. Strengths and Difficulties Questionnaire (SDQ)

The SDQ is a brief behavioral screening questionnaire. It is used to assess behavioral and emotional problems in children and adolescents and has good reliability and validity [38]. Internal consistency ranges from 0.60 to 0.78 across subscales. Test-retest reliability over 6 weeks is 0.79. There are 25 common items, and each item is rated on a 0 to 2 scale, 0: does not meet, 1: somewhat meets, and 2: fully meets, with five items, 7, 11, 14, 21, and 25, being reverse scored. The impact factor consisted of two items, “distress to the child” and “social deficits caused to the child,” which were scored on a 0-2 scale, both of which were positive. It resulted in the assessment of five factors: emotional symptoms, conduct problems, hyperactivity, peer interaction problems, and prosocial behavior, and a total difficulty score, which was composed of emotional symptoms, conduct problems, hyperactivity, and peer interaction problems.

#### 2.4.3. Questionnaire-Children with Difficulties (QCD)

The QCD can be used to assess the causal difficulties faced by children at specific time periods throughout the day and has been tested in four medical centers in Japan and China [39]. The Chinese version of the QCD has been shown to have good reliability and validity in Chinese children and adolescents with ADHD. Cronbach's alpha for QCD was 0.88. A total of 20 questions were designed with different subdimensions according to the time periods of the whole day, which was divided into morning/before school, during school, after school, evening, night/before bedtime, and overall performance, with four score options for each question and four levels of “0=totally disagree, 1=partially agree, 2=basically agree, 3=totally agree.” The sum of the scores for the questionnaire as a whole and each subdimension represented the child's performance at different times. The higher the score indicates higher life functioning and less difficulty the child had in daily related activities at that time.

### 2.5. Research Process

**Figure 3:**
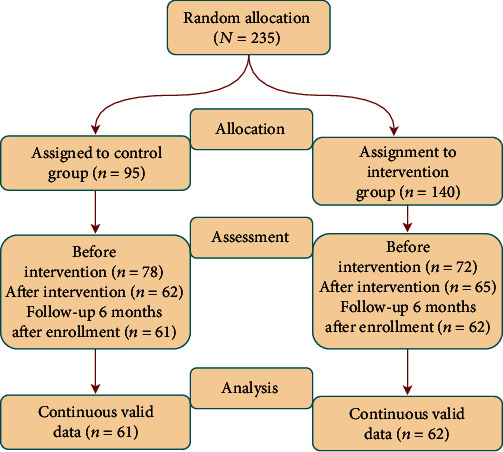
Process for the group training of parents of preschool children with attention deficit hyperactivity disorder.

After randomization and before the start of the intervention, 68 families in the intervention group did not participate in any sessions due to closures caused by the epidemic and parental time coordination difficulties, and 17 families in the control group dropped out of the program due to the epidemic after baseline assessment. Seven families in the intervention missed more than three sessions. A series of measures were taken to better motivate parents to participate in the sessions, including establishing group contacts to keep in touch with parents and help answer their questions, providing timely reinforcement during the sessions for parents who attended and completed assignments on time, and encouraging them to communicate with other family members about the intervention content after the sessions to gain family support. In addition, encouraging both parents to participate in the sessions also largely avoided the problem of absenteeism due to one of the parents being absent due to a temporary commitment, with 90% of the families ultimately missing at most one session or fully participating in all eight sessions. Families enlisted in the control cohort were not engaged in the programmatic intervention. However, they were furnished with fundamental psychoeducational resources pertinent to ADHD, as deemed necessary. At postintervention follow-up, two families in the intervention group did not participate in the follow-up assessment due to postpromotion class schedule conflicts, and one family dropped out of the follow-up assessment because of to a closure due to the epidemic. One family in the control group withdrew from the follow-up assessment.  Figure 3 depicts the details of the subjects.

### 2.6. Treatment

System-based parent group therapy is an intervention program for preschool ADHD implemented by parents in the home's natural environment. This parent group therapy is based on a systemic theory that considers problem behaviors in the child and in others in the environment, primarily, the primary caregivers, i.e., the parents. Therefore, the parental implementation of interventions to change the child's behavior is a two-way interactive process. Thus, the design of an intervention program needs to consider the influencing factors of both parents and children. These include the susceptibility factors of both parents, as well as some of the thoughts and feelings of the parents prior to the triggering event, and the influence of the thoughts and feelings of both parents and children on each other's behavior. Therefore, the program trains parents to master a systemic perspective on their child's behavior, to understand it, and to change it by adjusting parent-child interactions and creating a supportive environment.

The systemic-based parent group therapy is led by two trained facilitators, with all participating parents meeting on a fixed schedule once a week. The curriculum primarily encompasses five major themes: parent-child relationship, emotion management, behavior management, environment setting, and time management. The program comprises eight consecutive sessions, with one session per week, each lasting 1.5 hours, described in Table 1.

In session 1, we first introduce the basic principles and logic of the course, engage in team building, present the fundamentals and characteristics of ADHD, and explain the patterns of parent-child interaction and the first skill of the course: positive attention. The practice of positive attention is conducted through special parent-child time activities, which include several key skill strategies, succinctly referred to as “Three Don'ts and Four Dos.” The “Three Don'ts” are “Don't give orders, don't ask questions, don't judge,” while the “Four Dos” are “Describe, Imitate, Give feedback, Praise.” This skill is drilled on-site through role-playing. At the end of the course, a typical case study quiz is used to allow parents to review and consolidate the key skills of the lesson, and homework is assigned for parents to continue practicing at home, including filming a 5-10 minute video utilizing positive attention skills. The positive attention skill runs through the entire intervention process and is also the homework that parents must do for each class.

From session 2 to session 8, the course is structured as follows: feedback on homework (20-30 minutes), explanation of new skill strategies (30 minutes), on-site operation and practice (20-30 minutes), and typical case study quiz and assignment of homework (5-10 minutes). During the feedback session on homework, personalized, positive feedback is provided for each assignment, guiding and helping parents to improve their skills. Next, the principles and usage of new skill strategies are explained using cases provided by parents. On-site operations are often performed through role-playing. In the final segment, a quiz featuring 3-5 cases of nonstandard skill usage is used to help parents further consolidate and apply new skill strategies and also help therapists understand the parents' mastery of the skills. Homework includes video homework and paper homework, emphasizing communication and collaboration with other family members in using course skills.

Session 2 explains the behavior function analysis, making parents realize that parent-child interaction is a dynamic, mutually influencing system, rather than a one-sided cause-and-effect relationship. The main skills include behavior patterns and behavior recording, reinforcement and reward strategies, and verbal praise.

Session 3 introduces the methods of direct behavioral function analysis. Through video observation and feedback, parents can see their direct impact on their children's behavior as part of the ABC theory, improve self-awareness, and change capability, further reducing parent-child conflicts at home. The main skill strategies include effective commands, using effective commands and positive attention strategies during activities. The specific skills of effective commands include finding the right time to give commands; gaining the child's attention; using specific, direct commands; issuing commands that match the facts; polite language; giving the child time to respond; full name tracking; and considering the child's actual ability, etc.

Session 4 introduces the principles of behavior increase, the process and techniques of making good behavior record charts, and the application of good behavior record charts at home. The main skill strategies include positive reinforcement, tokens, and behavior shaping.

Session 5 discusses the principles of handling difficult situations, parents' emotional management, and how to correctly ignore. The main topics include mindfulness, emotional awareness and acceptance, parental emotion regulation, relaxation and self-care, and ignoring strategies.

Session 6 continues from the results of the ABC behavior analysis theory, explaining punishment, corner time, and the implementation plan for corner time. The main skill strategies include effective punishment and corner time.

Session 7 introduces establishing routines for family life and activities. The main skill strategies include establishing routines, organization, visual cues, and preventing problems through structured antecedents.

In session 8, the main content includes time management, time management worksheets, and preparation for school. The main skill strategies include cultivating a sense of time, being the master of one's own time, tips for sparking motivation, dividing time nodes, effective timetables, managing homework time, and strategies for communication between home and school.

**Figure 4:**
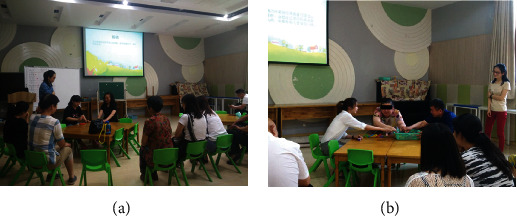
Parent training site: (a) scene 1 and (b) scene 2.

The parent training site is shown in Figure 4.

### 2.7. Statistical Treatment

Data analysis was conducted using SPSS version 23.0. The gender distribution was described in terms of counts (percentages), and the Chi-square test was used to compare the baseline gender distribution between the intervention and control groups. Normality of variables was assessed using the Shapiro-Wilk test, with normally distributed measures represented as mean ± standard deviation (*x̅*±*s*). Mauchly's test of sphericity was performed to validate the assumption of sphericity for repeated measures ANOVA. Where the assumption was violated, the Greenhouse-Geisser correction was applied to adjust degrees of freedom. Independent sample *t*-tests were utilized to compare baseline continuous demographic variables (age, IQ), as well as baseline scores on the ADHD-RS, SDQ, and QCD, between the groups. A two-way analysis of variance (ANOVA) was applied to examine the differences in symptom scores between the intervention and control groups across different genders. Both repeated measures analysis of covariance (ANCOVA) and repeated measures ANOVA were employed to analyze intragroup changes over time and intergroup differences in symptoms and functionality. After significant variance analysis, post hoc tests were conducted to determine changes in ADHD-RS, SDQ, and QCD scores at different specific time points within each group. To control the risk of type I errors, a Bonferroni correction was applied for multiple comparison adjustments. Differences were deemed statistically significant at a *P* value less than 0.05.

## 3. Results

### 3.1. Demographic Situation

Before the intervention, there were no significant differences between the intervention and control groups, with the exception of gender (all *P* > 0.05). An independent sample *t*-test was utilized to analyze the differences in scores between genders, and the results revealed no significant differences (all *P* > 0.05). Furthermore, a two-way analysis of variance (ANOVA) was conducted, with ADHD-RS, SDQ, and QCD scores as the dependent variables, and group and gender as fixed factors, to analyze the score differences between the intervention and control groups across different genders. The results indicated that only the main effect of gender was significant for the ADHD-RS total scores (*F* = 4.414, *P* = 0.038), suggesting significant differences between male and female assessment scores. However, the main effect of the group was not significant (*F* = 0.487, *P* = 0.487), and the interaction effect between gender and group was also not significant (*F* = 0.594, *P* = 0.442). This implies that the influence of gender on the ADHD-RS total scores is not dependent on the group. The demographics of the two groups are specified in Table 2.

### 3.2. Comparison of ADHD Core Symptoms between the Two Groups at Different Time Points after the Intervention

In the repeated measures ANCOVA, Mauchly's test indicated that the assumption of sphericity was met (*P* = 0.754). Therefore, sphericity was assumed, and no correction was applied. ADHD-RS total scores at times T0, T1, and T2 were considered within-subject factors, while the group (intervention or control) was treated as a between-subject factor. Sex was included as a covariate. At T0, the average ADHD-RS total scores for the intervention and control groups were 30.44 and 30.77, respectively. By T1, the average ADHD-RS score in the intervention group had decreased to 25.81, while the control group's score had slightly increased to 31.34. By T2, the intervention group's average ADHD-RS score had further declined to 24.82, while the control group's score remained relatively steady at 31.50.

The interaction of time and group was significant (Pillai's trace = 0.195, *F* = 14.385, *P* < 0.001), indicating a difference in ADHD-RS total scores between the intervention and control groups over time. However, the main effect of time was not significant (Pillai's trace = 0.001, *F* = 0.038, *P* = 0.962), implying that the ADHD-RS scores did not significantly change over time when the group factor was disregarded. The interaction of time and sex was also not significant (Pillai's trace = 0.011, *F* = 0.637, *P* = 0.531), suggesting no difference in ADHD-RS scores between males and females over time.

The within-subject effects test revealed that the interaction of time and group was significant (*F* = 15.302, *P* < 0.001), further demonstrating that the intervention had a differential effect on the change in ADHD-RS scores over time. The between-subject effects test showed that the effects of sex (*F* = 7.439, *P* = 0.007) and group (*F* = 4.618, *P* = 0.034) were significant. The ADHD-RS total scores for males in both groups were significantly higher than those of females (all *P*>0.05), but upon further analysis, the interaction between sex and group was not significant (*F* = 0.594, *P* = 0.442).

After eight consecutive weeks of training, The results of the repeated measures analysis of variance (ANOVA) revealed significant between-subject effects for the attention deficit score (*F* = 7.999, *P* = 0.005, partial *η*^2^ = 0.062). However, the between-subject effect was not significant for the hyperactivity-impulsivity score (*P* > 0.05). In the intervention group, there was a significant decrease in the total scores and subscale scores of the ADHD-RS scale (*F* = 17.425, *F* = 7.313, *F* = 18.035, *F* = 12.280, all *P* values < 0.001), indicating a statistically significant difference. However, in the control group, the decrease was not substantial, showing no statistically significant difference (*F* = 0.996, *F* = 0.746, *F* = 1.316, *F* = 0.327, all *P* values > 0.05). For details, see Table 3.

After a post hoc pairwise comparison with Bonferroni correction, the results showed that the total score on the ADHD-RS at T0 was significantly higher than at T1 (*P* < 0.001) and T2 (*P* < 0.001), while there was no significant difference between the scores at T1 and T2 (*P* = 0.310). The attention deficit score at T0 was significantly higher than at T1 (*P* < 0.05) and T2 (*P* < 0.05), but there was no significant difference between the scores at T1 and T2 (*P* = 0.581). The hyperactivity score at T0 was significantly higher than at T1 (*P* < 0.001) and T2 (*P* < 0.001), but there was no significant difference between the scores at T1 and T2 (*P* = 0.149). The impulsivity score at T0 was significantly higher than at T1 (*P* < 0.001) and T2 (*P* < 0.001), but there was no significant difference between the scores at T1 and T2 (*P* = 0.691).

### 3.3. Comparison of Symptoms between the Two Groups at Different Time Points after the Intervention

After the intervention and during the follow-up at six months postenrollment, the results from the repeated measures analysis of variance (ANOVA) showed that the differences between the groups were not statistically significant (*P* > 0.05). At postintervention and six-month follow-up of the group, the repeated measures ANOVA results showed that the SDQ scale, total scores, conduct problems, hyperactivity, and peer interaction scores significantly decreased in the intervention group, with statistically significant differences (*F* = 7.593, *F* = 3.355, *F* = 10.887, *F* = 4.626, all *P* < 0.05). The score for emotional symptoms in the intervention group did not significantly decrease from T0 to T1 (*P* > 0.05), but it did significantly decrease from T0 to T2 (*P* = 0.037). The prosocial behavior score in the intervention group did not significantly decrease (all *P* values > 0.05). The total score and scores for each factor in the control group did not significantly decrease (*F* = 0.428, *F* = 0.164, *F* = 1.066, *F* = 1.733, *F* = 0.187, *F* = 0.803, all *P* values >0.05). For details, see Table 4.

### 3.4. Comparison of Daily Social Functioning at Different Time Points before and after Intervention in Both Groups

At postintervention and six-month follow-up of enrollment, the repeated measures ANOVA showed that the total QCD scale score and each factor score increased significantly in the intervention group, with statistically significant differences (*F* = 6.746, *F* = 4.872, *F* = 5.983, *F* = 5.107, *F* = 6.726, *F* = 5.806, all *P* < 0.05); in the control group, the increase was not significant, and the differences were not statistically significant (*F* = 0.728, *F* = 0.335, *F* = 1.415, *F* = 2.357, *F* = 1.931, *F* = 1.142, all *P* > 0.05). But when comparing the total QCD scores and factor scores of the intervention group and the control group at the end of the treatment, the between-subject effects were not significant (all *P* values > 0.05). For details, see Table 5.

## 4. Discussion

In this multicenter, randomized controlled study, we investigated the therapeutic effects on symptoms of ADHD in preschool-aged children through a parent group based on systems theory emphasizing bidirectional parent-child interactive behavior analysis. Compared to the control group that did not receive parental group intervention, our research data indicates that after participating in the parent group for 8 weeks, with 1.5-hour sessions once a week, the core symptoms of ADHD in affected children, especially inattention, were significantly improved. According to the within-group effect analysis results, the parent group was able to significantly improve ADHD's emotional symptoms, conduct problems, hyperactivity symptoms, and peer interaction issues at the end of the 2-month intervention. It also enhanced daily life functioning and reduced difficulties encountered in the learning process. Moreover, the therapeutic effects were maintained to a certain extent during the 6-month follow-up period after enrollment, particularly in the improvement of emotional symptoms.

According to the statistical results after the intervention and follow-up in this study, the group that received parent training intervention showed significant improvement in ADHD-RS scores (*P* < 0.05) compared to the control group that did not receive intervention. This supports the study's hypothesis that parent training based on a systemic view of parent-child interaction can improve the symptoms of children with ADHD. Parent training may improve core symptoms by enhancing parents' behavior management skills, adjusting parent-child interaction patterns, helping parents create a supportive environment, and promoting self-regulation of emotions and behavior in children with ADHD. Specifically, parent training teaches parents to describe, imitate, provide positive feedback, and praise the child, enhancing positive parent-child interaction. Parents also better understand the mutual influence of parent-child behavior through behavior function analysis based on parent-child interaction, thus better coping with child behavior. Additionally, parents learn to appropriately use praise and rewards to enhance child behavior. The application of these strategies reduces unnecessary conflicts between parents and children and helps children establish self-regulation. In terms of emotion management, parent training enhances parents' awareness of their children's and their own emotions, helping parents maintain rational thinking when dealing with children. This not only helps parents adopt more appropriate strategies but also provides children with a model to enhance their emotional regulation abilities. In summary, parent training improves parents' behavior management skills, adjusts parent-child interaction patterns, helps parents create a supportive environment, and promotes the enhancement of self-regulation and adaptability in children with ADHD, objectively reflected in the core symptom scale, achieving the effect of alleviating ADHD symptoms. The research results fully validate the positive role of parent training based on a systemic view in treating preschool ADHD.

Based on the changes in SDQ and QCD scores, we observed significant functional improvements in children with ADHD as reported by parents after parent group sessions. However, the improvements in the intervention group in terms of QCD and SDQ were not significant in the variance analysis, and one potential reason for this finding is that this may be related to the impact of the pandemic. During the pandemic, many schools sporadically suspended offline courses and switched to online classes at home, disrupting the usual school routine. Children with ADHD faced more challenges and maladjustments in learning, emotions, behavior, and social interactions [40, 41]. Conflicts between parents and children or among siblings were more likely to occur when children with ADHD were studying at home, and the time for children to engage in extracurricular activities with peers was also greatly reduced. These factors might be the main reasons for the nonsignificant difference in functional improvement between the intervention group and the control group. At the same time, this may also suggest that, under the special circumstances of the pandemic, merely conducting parent group interventions may not be sufficient. More targeted intervention measures may be needed, such as including children in the intervention, simultaneously conducting child group sessions, or implementing interventions related to children's regular home learning routines, etc.

Emotional difficulties are common in children with ADHD [42], and there is a bidirectional relationship between parent-child emotional behavior. The higher the parents' enthusiasm, the less the child's withdrawal/depressive behavior; the more the parents criticize, the greater the child's aggressiveness [43]. In this study, the intervention group showed a declining trend in emotional symptom scores after the intervention, and this was well maintained at a 6-month follow-up after enrollment. According to systems theory, children's behavior is not seen as an isolated element but as part of a bidirectional interaction between parent and child. When one party in the parent-child interaction changes, the other may also change. Based on this, training parents in emotional management skills helps change themselves, and as parents make adjustments, the other party in the interaction, i.e., the child, will respond accordingly, thereby helping and improving the emotional regulation and management skills of children with ADHD. Moreover, children with ADHD have serious social impairments [44], which are related to their emotional difficulties [45]. Studies have reported that negative emotions or emotional intensity in children with ADHD in frustrating tasks are related to peer or parental reports of peer rejection [46]. After the intervention, there was some improvement in the prosocial behavior of children with ADHD, but unfortunately, the effect was not further maintained at a 6-month follow-up after enrollment. We hypothesize that the lack of good maintenance of prosocial behavior is influenced by various factors such as the child's temperament, personality, and social environment [47, 48], which may not be easily changed by parental training alone. Furthermore, these factors may require a longer time and more intensive intervention to show significant effects. We acknowledge the limitations of this study and suggest that future research should explore the mechanisms and moderating factors of parental training on the prosocial behavior of preschool children with ADHD.

Overall, this study, grounded in systems theory, proposes a training intervention method for preschool ADHD children based on parent-child interaction. Systems theory emphasizes the analysis of mutual interactions and bidirectional influences between parents and children, rather than viewing behavior as the product of isolated factors. Within the context of parent-child interaction, the perspective of systems theory focuses on how the behaviors of parents and children mutually influence each other in a continuous, interrelated process. Our research findings support this view, indicating that enhancing parental behavior management skills can positively affect the symptoms of children with hyperactivity disorder. Parent training enables parents to implement more effective behavior management according to the child's needs. In turn, this may help improve parent-child interaction, as well as the child's emotional regulation and symptoms of hyperactivity disorder. Our study demonstrates the practicality of the systems theory approach in conceptualizing and treating preschool ADHD by focusing on the interactions within parent-child relationships.

## 5. Conclusions

Although the long-term efficacy of this study has not yet been provided, this preliminary research offers initial evidence, demonstrating the prospects of a parent training program in effectively improving the core ADHD symptoms and emotional behavior and social functioning in preschool children, without any safety issues identified so far. However, since preschool ADHD usually requires very cautious medication treatment and poses safety concerns, this preschool parent group may fill the gap in China's preschool ADHD parent behavior management training, to avoid using medication as the first-line treatment option. Notably, the efficacy of the intervention group continues three months after the intervention, indicating that the effects will be further maintained, and a virtuous cycle begins once the parents master the skills. Lastly, compared to individual treatment, the parent group is a cost-effective intervention option. However, the conclusion is affected by a high dropout rate due to the pandemic, resulting in a small sample size and limitations in assessment measures, so there is still a need for a larger sample and more comprehensive and objective assessment measures (such as independent observations, audiotapes) for long-term follow-up to further validate these preliminary findings. In the future, the synergistic enhancement effect can be judged by comparing combined treatment (parent training + medication) with medication alone. Simultaneously, the intervention plan will be expanded to directly involve children, in conjunction with parent training, and, through longitudinal research, further determine whether early intervention brings sustained benefits in academic and social functions.

## Figures and Tables

**Table 1:** The themes and main intervention strategies of parent group therapy on the systemic theory.

Session no.	Topics	Key strategies
Session 1	Parent-child relationship	Positive focus
Session 2	Behavior management	Behavior analysis
Session 3	Parent-child relationship	Effective command; movement
Session 4	Behavior management	Positive reinforcement; behavior shaping
Session 5	Emotion management	Emotional awareness, acceptance; emotion regulation neglect
Session 6	Behavior management	Consequences of behavior; corner time
Session 7	Environment settings	Organizing; structuring; visual cues
Session 8	Time management	Time management

**Table 2:** Comparison of demographic information and ADHD-RS, SDQ, and QCD scores between the intervention and control groups.

Projects	Intervention group	Control group	Cardinality/*t* value	*P*
Gender (male/female, example)	49/13	56/5	4.014	0.045
Age (years, *x* ± *s*)	4.92 ± 0.59	5.12 ± 0.62	-1.791	0.076
IQ (*x* ± *s*)	101.05 ± 13.91	99.74 ± 14.82	0.506	0.614
Parental education				
Junior high school and below	3	10	7.003	0.136
High school/junior high school/technical school	7	11		
College	23	20		
Undergraduate	24	18		
Master and above	5	2		
Parental occupation				
Government civil servants	0	2	7.036	0.533
Science, education, culture, health, and other professionals	9	7		
Corporate management	4	5		
Enterprise workers/workers	12	8		
Self-employed	7	5		
Freelancer	22	19		
Farmers/migrant workers	1	4		
Others: none	7	1		
Annual household income				
Under 50,000 ¥	2	9	8.707	0.069
50-100,000 ¥	18	18		
100-200,000 ¥	22	23		
200,000-500,000 ¥	14	10		
500,000 ¥ or more	6	1		
ADHD-RS				
Summary table score	30.44 ± 9.65	30.77 ± 10.45	-0.179	0.858
Attention to defects	14.82 ± 4.94	15.90 ± 6.56	-1.026	0.307
More movement	10.85 ± 4.28	10.33 ± 4.12	0.681	0.497
Impulse	4.77 ± 2.04	4.54 ± 2.51	0.564	0.573
QCD				
Total score	30.83 ± 8.85	33.46 ± 8.66	-1.667	0.098
Overall behavioral issues	2.81 ± 1.34	3.26 ± 1.39	-1.823	0.071
Early morning/before school	5.45 ± 2.53	5.98 ± 2.72	-1.106	0.271
School	5.13 ± 1.85	5.71 ± 2.00	-1.668	0.098
After school	5.32 ± 2.13	5.88 ± 2.28	-1.397	0.165
Evening	6.82 ± 2.46	7.02 ± 2.18	-0.471	0.639
Night	5.30 ± 2.23	5.62 ± 2.07	-0.840	0.403
SDQ				
Total score	17.19 ± 4.89	17.12 ± 5.21	0.073	0.942
Emotional symptoms	3.08 ± 1.97	2.93 ± 2.04	0.402	0.688
Character issues	3.76 ± 2.05	2.95 ± 2.45	1.976	0.050
More movement	7.85 ± 1.98	7.60 ± 2.25	0.648	0.518
Peer interaction issues	3.35 ± 2.11	3.36 ± 2.21	-0.022	0.982
Prosocial behavior	5.97 ± 2.00	6.45 ± 2.35	-1.236	0.219
Impact factor	5.01 ± 2.18	4.52 ± 1.90	1.315	0.191

Note: Differences were considered statistically significant at *P* < 0.05.

**Table 3:** Comparison of ADHD-RS scores at different time points in the intervention and control groups ($\bar{x}\pm \text{s}$).

	Total score		Attention deficit		Hyperactivity		Impulse	
	Intervention group	Control group	Intervention group	Control group	Intervention group	Control group	Intervention group	Control group
T0	30.44 ± 9.65	30.77 ± 10.45	14.82 ± 4.94	15.90 ± 6.56	10.85 ± 4.28	10.33 ± 4.12	4.77 ± 2.04	4.54 ± 2.51
T1	25.81 ± 9.20	31.34 ± 8.92	13.07 ± 4.41	16.24 ± 5.78	8.98 ± 4.14	10.64 ± 3.89	3.75 ± 2.24	4.45 ± 2.34
T2	24.82 ± 9.58	31.50 ± 9.75	12.77 ± 4.97	16.27 ± 6.14	8.40 ± 4.02	10.72 ± 4.22	3.65 ± 2.06	4.51 ± 2.32
*F*	17.425	0.996	7.313	0.746	18.035	1.316	12.280	0.327
*P*	<0.001	0.372	0.001	0.476	<0.001	0.276	<0.001	0.722

Note: T0: baseline period; T1: 2 months after enrollment; T2: 6-month follow-up after enrollment. *F* and *P* values are for within-group repeated measures ANOVA across timepoints, with *P* < 0.05 as a statistically significant difference. Higher ADHD-RS scores indicate greater severity.

**Table 4:** Comparison of SDQ scores at different time points in the intervention and control groups ($\bar{x}\pm \text{s}$).

SDQ	T0		T1		T2		*F*/*P*	
	Intervention group	Control group	Intervention group	Control group	Intervention group	Control group	Intervention group	Control group
Total score	17.19 ± 4.89	17.12 ± 5.21	15.01 ± 6.11	17.24 ± 5.33	12.63 ± 19.12	16.78 ± 7.20	7.593/0.001	0.428/0.654
Emotional symptoms	3.08 ± 1.97	2.93 ± 2.04	2.59 ± 2.07	2.96 ± 2.06	1.74 ± 5.41	2.76 ± 3.52	2.529/0.088	0.164/0.849
Conduct problems	3.76 ± 2.05	2.95 ± 2.45	2.91 ± 1.87	3.23 ± 2.12	2.75 ± 5.51	3.20 ± 2.14	3.355/0.042	1.066/0.348
Hyperactivity	7.85 ± 1.98	7.60 ± 2.25	6.79 ± 2.50	7.70 ± 2.10	5.53 ± 5.22	7.27 ± 2.88	10.887/<0.001	1.733/0.186
Peer interaction problems	3.35 ± 2.11	3.36 ± 2.21	2.72 ± 1.69	3.35 ± 2.13	2.61 ± 7.43	3.56 ± 3.78	4.626/0.014	0.187/0.830
Prosocial behavior	5.97 ± 2.00	6.45 ± 2.35	6.66 ± 2.61	6.53 ± 2.13	6.41 ± 2.61	6.62 ± 2.57	2.001/0.140	0.803/0.453
Impact factor	5.01 ± 2.18	4.52 ± 1.90	4.24 ± 1.90	4.54 ± 1.79	3.98 ± 1.86	4.29 ± 1.65	9.199/<0.001	0.950/0.332

Note: T0: baseline period; T1: 2 months after enrollment; T2: 6-month follow-up after enrollment. *F* and *P* values are for within-group repeated measures ANOVA; *P* < 0.05 as a statistically significant difference.

**Table 5:** Comparison of QCD scores at different time points in the intervention and control groups ($\bar{x}\pm \text{s}$).

		T0	T1	T2	*F*	*P*
Total score	Intervention group	30.83 ± 8.85	36.43 ± 7.84	36.09 ± 12.06	11.633	<0.001
	Control group	33.46 ± 8.66	33.87 ± 8.43	34.13 ± 8.32	0.995	0.376

Overall behavior	Intervention group	2.81 ± 1.34	3.55 ± 1.32	3.48 ± 2.02	6.746	0.002
	Control group	3.26 ± 1.39	3.31 ± 1.33	3.19 ± 1.36	0.728	0.487

Early morning/before going to school	Intervention group	5.45 ± 2.53	6.71 ± 2.66	7.11 ± 4.60	4.872	0.011
	Control group	5.98 ± 2.72	5.91 ± 2.72	6.01 ± 2.56	0.335	0.717

School	Intervention group	5.13 ± 1.85	5.90 ± 1.75	5.44 ± 2.37	5.983	0.004
	Control group	5.71 ± 2.00	5.68 ± 1.88	5.38 ± 2.07	1.415	0.251

After school	Intervention group	5.32 ± 2.125	6.16 ± 1.94	5.81 ± 2.22	5.107	0.007
	Control group	5.88 ± 2.28	6.05 ± 2.22	6.01 ± 2.44	2.357	0.104

Evening	Intervention group	6.82 ± 2.46	7.91 ± 2.22	7.90 ± 3.76	6.726	0.002
	Control group	7.02 ± 2.18	7.14 ± 2.19	7.65 ± 3.22	1.931	0.154

Night	Intervention group	5.30 ± 2.23	6.20 ± 1.90	6.36 ± 3.02	5.806	0.005
	Control group	5.62 ± 2.07	5.78 ± 2.15	5.89 ± 2.39	1.142	0.326

Note: T0: baseline period; T1: 2 months after enrollment; T2: 6-month follow-up after enrollment. *F* and *P* values are for within-group repeated measures ANOVA; *P* < 0.05 as a statistically significant difference. Higher QCD scores indicate better functioning.

## Data Availability

All data used to support the findings of this study are included within the article.
